# A Systematic Review of the Reporting Quality of Observational Studies That Use Mediation Analyses

**DOI:** 10.1007/s11121-022-01349-5

**Published:** 2022-02-15

**Authors:** Rodrigo R. N. Rizzo, Aidan G. Cashin, Matthew K. Bagg, Sylvia M. Gustin, Hopin Lee, James H. McAuley

**Affiliations:** 1 School of Health Sciences, University of New South Wales, Sydney, Australia; 2 Centre for Pain IMPACT, Neuroscience Research Australia, Sydney, Australia; 3 Prince of Wales Clinical School, University of New South Wales, Sydney, Australia; 4 New College Village, University of New South Wales, Sydney, Australia; 5 School of Psychology, University of New South Wales, Sydney, Australia; 6 Centre for Statistics in Medicine, Nuffield Department of Orthopaedics Rheumatology and Musculoskeletal Sciences (NDORMS), University of Oxford, Oxford, UK; 7 School of Medicine and Public Health, University of Newcastle, Newcastle, Australia

**Keywords:** Mechanism, Mediation analysis, Systematic review, Observational studies, Reporting, Publication, Prevention

## Abstract

Mediation analysis is a common statistical method used to investigate mechanisms of health exposure and interventions. The reporting quality of mediation studies used in randomised controlled trials has been considered heterogeneous and incomplete. The reporting quality of mediation analysis in observational studies is unknown. We conducted a systematic review to describe the reporting standards of recently published observational studies that used mediation analysis to understand the mechanism of health exposures. We searched for studies published between June 2017 and June 2019 indexed in EMBASE, MEDLINE and PsycINFO. Two reviewers screened articles and selected a random sample of 50 eligible studies for inclusion. We included studies across 13 healthcare fields and ten different health conditions. Most studies (74%) collected data on healthy individuals to assess their risk of developing a health disorder. Psychosocial and behavioural factors (self-control, self-esteem, alcohol consumption, pain) were the most prevalent exposures (*n* = 30, 60%), outcomes (*n* = 23, 46%) and mediators (*n* = 29, 58%). Most studies used a cross-sectional design (64%, *n* = 32), and a few studies reported sample size calculations (4%, *n* = 8). In 20% (*n* = 10) of the studies, adjustment for confounders was reported. Only 10% (*n* = 5) of studies reported the assumptions underlying the mediation analysis, and 14% (*n* = 7) of studies conducted some sensitivity analysis to assess the degree which unmeasured confounders would affect the estimate of the mediation effect. Mediation analysis is a common method used to investigate mechanisms in prevention research. The reporting of mediation analysis in observational studies is incomplete and may impact reproducibility, evidence synthesis and implementation.

**Supplementary Information:**

The online version contains supplementary material available at 10.1007/s11121-022-01349-5.

## Introduction

Mediation analysis is a common statistical method used to investigate mechanisms of prevention strategies (Mackinnon & Dwyer, 1993; MacKinnon et al., 2002). In mediation analysis, the total effect of an exposure on an outcome is separated into an “indirect effect” that works through a hypothesised mediator(s), and a “direct effect”, which is the effect of the exposure on the outcome that is not explained by the mediator(s) under study (MacKinnon & Pirlott, 2015). A typical mediation analysis includes an exposure, mediator, outcome and confounders of the exposure-mediator, exposure-outcome and mediator-outcome effects.

The information gained from mediation analyses used in observational studies can inform policy decisions, lead to intervention optimization and guide implementation (Mackinnon & Fairchild, 2009; Moore et al., 2015). For example, Huang et al. (2016) conducted a mediation analysis of an observational cohort and found that personality traits affected mental health partly through its effect (indirect effect) on sleep quality. This study informed the design of preventive programs to improve mental health through targeting sleep quality in randomised controlled trials (Freeman et al., 2017; Waite et al., 2020). Others have investigated the role of peer influence on the effect of drug exposure on drug use (Rudolph et al., 2018; Studer et al., 2014). For this reason, several interventions commonly include preventive approaches to improve the skills of adolescents and adults to deal with peer influence (Birrell et al., 2021; McCormack, 2021).

Historically, there have been two approaches in mediation analyses: traditional approaches and modern approaches that incorporate causal inference principles (i.e., causal mediation approaches) (Nguyen et al., 2019; VanderWeele, 2016). The traditional approaches of mediation analysis refer to path analysis method (e.g. structural equation modelling (Wright, 1931), causal steps method (i.e. Baron and Kenny method) and methods that estimate the indirect effect using the product-of-coefficients and the difference-of-coefficients methods (MacKinnon et al., 2007). In these approaches, the mediated effect is estimated using regression models (MacKinnon et al., 2007). For example, the Baron and Kenny method uses a sequence of significance tests to determine the presence of a mediated effect (Baron & Kenny, 1986). The product-of-coefficients method estimates the indirect effect multiplying the exposure-mediator coefficient by the mediator-outcome coefficient of the regression analyses (MacKinnon et al., 2007; VanderWeele, 2016). Causal mediation approaches propose non-parametric definitions of the effects, clarify causal assumptions required for these effects, use techniques to scrutinise these assumptions based on principles of causal inference, and accommodate more realistic settings including non-linear relationships and exposure-mediator interactions (Imai et al., 2010; Nguyen et al., 2019).

Despite increased popularity and advances of mediation analyses over the past 15 years (Nguyen et al., 2019), reporting quality has been inconsistent and incomplete across different disciplines, study designs and publication types (Cashin et al., 2019; Vo et al., 2019). Systematic reviewers have expressed difficulty in synthesising the results of mediation studies because of inadequate reporting of effect sizes, precision estimates and statistical analysis techniques used in the studies (Cashin et al., 2019). Most randomised controlled trials do not adjust the mediation analysis for potential confounders (Vo et al., 2019). Observational studies have particular challenges that may influence the reporting of the study, such as the reporting of confounders for the exposure-mediator and exposure-outcome effects and more flexibility in choosing the time points of the assessments (Valente et al., 2017). The standard of reporting of observational studies is still uncertain. Previous systematic reviews limited their inclusion criteria to a particular method of mediation analysis or a specific type of observational design. Gelfand et al. (2009) included observational studies using the Baron and Kenny approach, while Liu et al. (2016) included studies that used the counterfactual framework approach for mediation analysis, and Lapointe-Shaw et al. (2018) limited the inclusion to time-to-event outcomes.

This systematic review aims to describe the standard of reporting of published observational studies that used mediation analysis to understand the mechanisms of health exposures.

## Methods

### Study Design

This systematic review is reported in accordance with the Preferred Reporting Items for Systematic Review and Meta-analyses (PRISMA) guideline (Liberati et al., 2009). The review was prospectively registered on PROSPERO (CRD42019136348). Protocol deviations are reported in Online Resource 1.

### Search and Selection of Studies

We developed a search strategy to identify studies that used mediation analysis to understand the mechanisms of health exposures (Online Resource 2). We searched EMBASE, MEDLINE and PsycINFO, through Ovid, to identify records published between June 30, 2017 and June 30, 2019. We limited the inclusion criteria to mediation studies published between 2017 and 2019 to understand the standard of mediation studies before the implementation of A Guideline for Reporting Mediation Analyses of Randomized Trials and Observational Studies (The AGReMA Statement) (Lee et al., 2021) that was expected to be published in 2020 or 2021. We exported the references retrieved from the search database into an *Excel* workbook, removed duplicates and generated a random number for each record. Consecutive sets of 15 records (titles, abstracts and full texts) were screened in duplicate until both reviewers (RNRR and AGC) were in complete agreement for an entire set. Then, the screening was performed by one reviewer (RRNR) until 50 records were included. We included a sample of 50 studies following previous systematic reviews that informed the development of reporting guidelines (Phillips et al., 2014; Tooth et al., 2005) and other systematic reviews that assessed the reporting quality of mediation studies in observational designs (Gelfand et al., 2009).

### Eligibility Criteria

We included observational studies that applied mediation analysis to understand the mechanisms of health exposures. We made no restriction on the type of mediation analysis, health condition, exposure, mediator or outcome. We included primary studies where the data were collected for the purpose of conducting the mediation analysis; and secondary studies where mediation analysis was applied to data collected for other purposes. We excluded reports of randomised exposures (or interventions), systematic reviews, protocols, non-English and articles for which full texts were not available after several attempts using search engines and academic repositories.

### Data Extraction

We developed a data extraction form in REDCap (Harris et al., 2009). Two reviewers (RRNR and AGC) pilot tested the extraction form on a sample of 12 studies. Then, 10% of the included studies were extracted by two reviewers, and the remaining 90% were extracted by one reviewer (RRNR). Double extraction was used to assess the inter-rater agreement between reviewers. We extracted study characteristics related to publication (publication year, journal), design (cross-sectional, longitudinal, retrospective, prospective), primary or secondary study, sampling (health condition, number of participants) and measurement (number, type, measure and time point for the exposure(s), outcome(s) and mediator(s)). We categorized the health conditions (e.g. mental disorders, neurological disorders, substance abuse) based on the World Health Organization definition (WHO, 2019). We assessed the presence or absence of several items related to the standard of reporting of mediation analysis of observational studies described in the Online Resource 3. The assessment included items based on previous reviews and existing methodological and reporting guidance (Cashin et al., 2019; Cerin & MacKinnon, 2009; Fairchild & McQuillin, 2010; Gelfand et al., 2009; Hertzog, 2018; Imai & Yamamoto, 2013; Kraemer et al., 2002; Lange et al., 2017; MacKinnon et al., 2012; Mansell et al., 2013; Mascha et al., 2013; VanderWeele, 2015; Wood et al., 2008). Briefly, we included items to understand whether the mediation studies reported relevant terms for mediation analysis in the title and abstract, provided the rationale for studying mediation in the context of the study and described details about the methods (e.g. confounders, causal assumptions, statistical methods and measurements) and reported the different effects in the “Results Section” of the study (e.g. total, direct and indirect effect and sensitivity analysis).

Reporting of these items has been considered essential to appropriately interpret, reproduce and apply the findings from studies that use mediation analysis. We included most of the items reported in the recently published AGReMA Statement (Lee et al., 2021). In the Online Resource 3, we indicated the items included in our review that are also included in the AGReMA Statement.

### Data Synthesis and Analysis

We summarised each data item with frequencies and percentages for categorical variables, and median and interquartile range for continuous variables. We analysed data using Microsoft Excel.

## Results

**Fig. 1:**
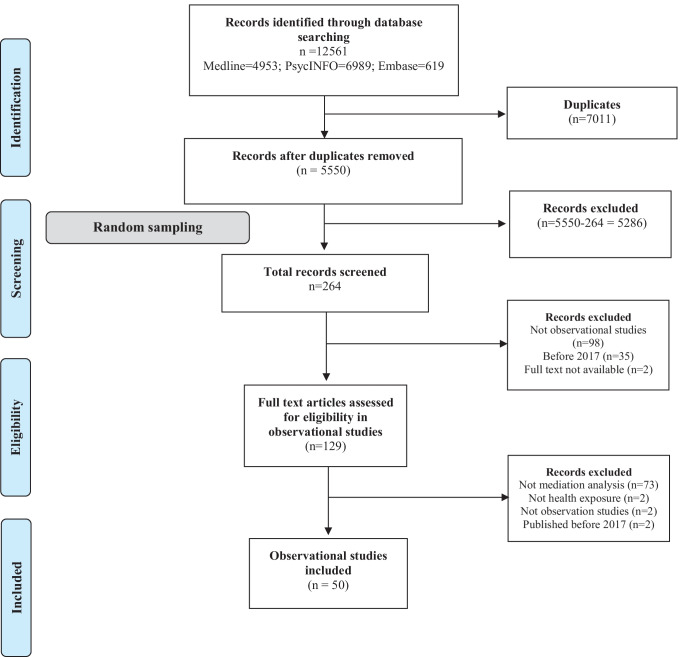
PRISMA flow diagram describing record screening and inclusion

The search identified 12,561 records. After removing duplicates, 5550 unique records were identified. The unique records were randomised and then screened following our eligibility criteria. We reached a complete agreement for study inclusion in the first set of 15 consecutive records. A reviewer (RNRR) screened 264 titles and abstracts. From 264 records, 129 full-text records were identified as potentially eligible studies. After the full-text screening, we included the desired sample of 50 included records for data extraction (Fig. 1). The agreement between the two reviewers (RRNR and AGC) from extraction (10% of the sample) was 0.76 (Kappa coefficient), which represents substantial agreement (Rigby, 2000).

## General Characteristics of Included Studies

We listed the included studies in Online Resource 4. Among 50 included studies, 58% (*n* = 29) were published in 2017, 38% (*n* = 19) in 2018 and 4% (*n* = 2) published in 2019. The most common study design was cross-sectional (64%, *n* = 32), followed by cohort (28%, *n* = 14) and retrospective studies (4%, *n* = 8). Primary data were used for 36% of studies (*n* = 18), and secondary data for 64% of studies (*n* = 32). The included studies covered 13 healthcare fields, and healthy participants were included in 74% of studies (*n* = 37) (Table 1).

**Table 1:** General characteristics of the included studies

Characteristics (*n* = 50)	
**Study stage, *n* (%)**	
Primary study	18 (36)
Secondary analysis of a previous study or existing database	32 (64)
**Study design, *n* (%)**	
Cross-sectional	32 (64)
Longitudinal retrospective	4 (8)
Cohort	14 (28)
**Broad approach for mediation analysis, *n* (%)**^**c**^	
Traditional	45 (90)
Causal mediation analysis (using counterfactual approach)	5 (10)
**Number of participants in the mediation model, median (IQR)**	456 (214–1264)
**Healthcare field, *n* (%)**	
Mental Health	19 (38)
Substance abuse	6 (12)
Behavioural medicine	5 (10)
Aging	4 (8)
Diabetes and obesity	3 (6)
Public health	3 (6)
Cardiology	2 (4)
Occupational health	2 (4)
Other	6 (12)
**Health condition of participants, *n* (%)**	
Healthy participants	37 (74)
Mental disorders	3 (6)
Neurological disorders	2 (4)
Pain	2 (4)
Substance abuser	2 (4)
Other	4 (8)
**Number of exposures studied, *n* (%)**	
Single exposure	36 (72)
Multiple exposures	14 (28)
**Type of exposure, *n* (%)**	
Psychosocial and behavioural^a^	30 (60)
Symptom of a mental health^b^	9 (18)
Demographic	5 (10)
Biological	4 (8)
Physical function	2 (4)
**Number of outcomes studied, *n* (%)**	
Single outcome	35 (70)
Multiple outcomes	15 (30)
**Type of outcome, *n* (%)**	
Psychosocial and behavioural^a^	23 (46)
Symptom of a mental health^b^	16 (32)
Biological	7 (14)
Physical function	2 (4)
Cognition	2 (4)
**Number of mediators studied, *n* (%)**	
Single mediator	23 (46)
Multiple mediators	27 (54)
two mediators	14 (28)
three mediators	8 (16)
four mediators	2 (4)
five mediators	2 (4)
more than five mediators	1 (2)
**Type of mediators studied, *n* (%)**	
Psychosocial and behavioural ^a^	29 (58)
Symptom of a mental health ^b^	12 (24)
Biological	5 (10)
Cognition	3 (6)
Physical function	1 (2)
**Time point of the mediator, *n* (%)**	
Measured with the exposure and outcome	32 (64)
Measured after the exposure and before the outcome	12 (24)
Measured with the exposure	3 (6)
Measured with outcome	3 (6)

^a^Psychosocial and behavioural includes self-esteem, alcohol consumption, decision making, social connection, pain

^b^Symptom of a mental health includes stress, suicide, depression, anxiety

^c^Causal mediation analysis refers to reporting of the following terms in the study: causal mediation analysis or counterfactual framework

## Characteristics of the Exposures, Mediators and Outcomes

Psychosocial and behavioural factors (e.g. self-control, self-esteem, alcohol consumption, social connection, pain) were the most commonly investigated exposures (*n* = 30, 60%), outcomes (*n* = 23, 46%) and mediators (*n* = 29, 58%). Mental health symptoms (sleep disorder, stress, suicide and depression) were the second most prevalent exposures (*n* = 9, 18%), outcomes (*n* = 16, 32%) and mediators (*n* = 12, 34%) investigated in the mediation models. Multiple mediators were investigated in 54% of studies (*n* = 27). In 64% of studies (*n* = 32), the mediators and outcome were measured at the same time point (Table 1).

## Standard of Reporting in the Included Studies

### Title and Abstract

The mechanistic aim of the study (“mediation analysis” or “mechanism evaluation”) was reported in the title or abstract in 96% of studies (*n* = 48), and 48% of studies (*n* = 24) reported these key terms in both title and abstract (Table 2).

**Table 2:** Standard of reporting in the title, abstract introduction and methods of observational studies that used mediation analysis

Section/topic	Characteristic	*N* (%)
**Title and abstract**		
Did the articles report the mechanistic nature of the study? (Use of the following terms causality, mechanisms evaluation, indirect effect, and mediation analysis)		
Title and abstract		24 (48)
Abstract (only)		23 (46)
Title (only)		1 (2)
Not mentioned		2 (4)
**Introduction**		
Did the articles report the …?		
-Motivation for using mediation	Yes	49 (98)
-Rationale for studying mechanisms	Yes	49 (98)
-Intention of the study	Confirmatory	39 (78)
	Exploratory	6 (12)
	Unclear	5 (10)
Did the articles describe the …?		
-Hypothesis for mediation	Yes	33 (66)
-Rationale for the exposure-outcome relationship	Yes	46 (92)
-Rationale for the exposure-mediator relationship	Yes	46 (92)
-Rationale for the mediator-outcome relationship	Yes	48 (96)
**Methods**		
Did the articles report the …?		
-Protocol or registration	Yes	1 (2)
-Study design	Yes	11 (22)
-Main effect of the mediation study	Yes	33 (66)
-Graphical representation	Yes	38 (76)
Did the articles consider possible confounders in the mediation model?	Exposure-mediator or exposure outcome	21 (42)
	Mediator-outcome	14 (28)
	No confounders specified	10 (20)
	Unclear	19 (38)
Did the articles report the method to adjust for measured confounders?	Yes	13 (26)
Did the articles consider exposure-mediator interaction?	Yes	7 (14)
Did the articles explain how exposure-mediator interaction was modelled?	Yes	5 (10)
If relevant, did the articles specify the multilevel nature of the exposure, mediator, or outcome?	Yes	1 (2)
Did the articles specify the assumptions required for making causal inference?	No confounding (exposure-mediator)	5 (10)
	No confounding (exposure-outcome)	4 (8)
	No confounding (mediator-outcome)	4 (8)
	No exposure-dependent confounding (mediator-outcome)	
	No interactions	3 (6)
	Consistency	0 (0)
	Positivity	0 (0)
	No confounding (exposure-mediator)	0 (0)
Did the articles state *how* the exposure, mediator and outcome were defined and measured?	Yes	48 (96)
	No	1 (2)
	Partial (one or more missing)	1 (2)
Did the articles state *when* the exposure, mediator and outcome were defined and measured?	Yes	42 (84)
	No	6 (12)
	Partial (one or more missing)	2 (4)
Did the articles state how sample size was estimated for the mediation model?	Yes	4 (8)
Did the articles specify the statistical method to assess mediation?^a^	Yes	32 (64)
	Unclear (missing details to differentiate to other statistical methods)	4 (8)
Did the articles specify the statistical model to assess mediation?^b^	Yes	19 (38)
	Unclear (missing details to differentiate to other statistical models)	9 (18)
Did the articles mention the presence or absence of missing data?	Yes	25 (50)
Did the articles mention *how* missing data was handled?	Yes	25 (50)
Did the articles describe any approach to sensitivity analysis?	Yes	7 (14)
	Unclear (not clear if the intention of approach was related to do a sensitivity analysis)	3 (6)
Did the articles provide references to statistical software or packages used in the mediation analysis?	Yes	38 (76)

^a^Statistical method is defined as the statistical test used to assess mediation with references (e.g. the difference method, the product method, Baron and Kenny method, causal mediation)

^b^Statistical model is defined as the process of applying statistical analysis in the dataset (e.g. linear regression, logistic regression, Cox proportional hazards). The term “regression” only was checked as unclear

### Introduction Section

At least 92% of studies (*n* = 46) reported evidence or theory supporting a possible causal relationship between exposure, mediator and outcome. The exploratory or confirmatory nature of the study was reported in 90% of studies (*n* = 45) (Table 2).

### Methods Section

Only one study (2%) provided reference to a protocol or preregistration for the mediation study. A graphical representation of the tested model was reported in 76% of studies (*n* = 38). Only 38% of studies (*n* = 19) mentioned the statistical model used in the mediation analysis (e.g. difference-coefficient approach, Baron and Kenny’s framework, counterfactual-based approaches). Less than half of the studies considered possible exposure-mediator or exposure-outcome confounders (44%, *n* = 22) and mediator-outcome confounders (30%, *n* = 15). Sensitivity analysis for confounding was described in 14% of studies (*n* = 7). The assumptions required for making causal inferences were reported in 10% of the studies (*n* = 5). Other characteristics of the methods section are reported in Table 2.

### Results Section

Confidence intervals or standard errors for the total effect were provided in 60% of studies (*n* = 30). Precision for the direct effect was reported in 54% (*n* = 27), precision for indirect effect in 74% (*n* = 37), precision for exposure-mediator effect in (48% (*n* = 24) and precision for mediator-outcome effect in 50% of studies (*n* = 25). Other characteristics of the results section are reported in Table 3.

**Table 3:** Standard of reporting in the results section of observational studies that used mediation analysis

Section/topic	Characteristic	*N* (%)
**Results**		
Did the articles provide estimates for the …?		
Total effect	Effect	46 (92)
	Precision	30 (60)
	p value	38 (76)
Direct effect	Effect	36 (72)
	Precision	27 (54)
	p value	29 (58)
Indirect effect	Effect	46 (92)
	Precision	37 (74)
	p value	37 (74)
Exposure-mediator effect	Effect	44 (88)
	Precision	24 (48)
	p value	37 (74)
Mediator-outcome effect	Effect	24 (48)
	Precision	25 (50)
	p value	25 (50)
Proportion mediated	Effect	18 (36)
	Precision	2 (4)
	p value	3 (6)
Sensitivity analysis	Effect	7 (14)
	Precision	7 (14)
	p value	2 (6)

Total effect: the effect of the exposure on the outcome that encompasses all indirect and direct effects. Direct effect: the effect of the exposure on the outcome that is not explained by the mediator(s). Indirect effect: the effect of the exposure on the outcome that works through hypothesised mediator(s). Precision refers to the 95% confidence interval (95% CI)

## Discussion

This systematic review aimed to establish the reporting standards of observational studies that used mediation analysis to understand the mechanisms of health exposures. We assessed the reporting standards of 50 studies across 13 healthcare fields and ten different health conditions. Most studies (74%) assessed the risk of developing a health disorder among healthy participants. Psychosocial and behavioural factors (e.g. self-control, self-esteem, alcohol consumption, social connection, pain) were the most common variables used in mediation models.

The standard of reporting of mediation analysis in observational studies was incomplete and inconsistent. Less than half of the studies (48%) used terms “mediation analysis” or “mechanism evaluation” in the title and abstract, which may reduce the likelihood of mediation studies being identified in search strategies (Li et al., 2019).

In our sample, most studies did not clearly report how the exposure-outcome (58%) and the mediation-outcome (72%) confounding were addressed. Vo et al. (2019), showed that 57% of studies did not report adjustment for mediator-outcome confounders in randomised controlled trials. Without clearly reporting of which possible confounders were adjusted in the analysis, it is difficult to assess the risk of bias in studies that use mediation analyses (Valente et al., 2017; VanderWeele & Chiba, 2014). Sensitivity analyses are encouraged to assess the robustness of the study to confounding bias (VanderWeele & Chiba, 2014). However, in our review, only 14% of the studies reported such sensitivity analyses.

In most mediation analyses, it is expected that the exposure precedes the mediator, and that mediators precede the outcome (Gelfand et al., 2009; Mansell et al., 2013). A common method to ensure temporal precedence is through longitudinal assessments (Gelfand et al., 2009; Mansell et al., 2013; Rizzo et al., 2021). However, longitudinal assessments of the exposure, mediator(s) and outcome alone do not necessarily guarantee a causal order and instead, also requires a plausible theoretical explanation about the direction of effects. For example, Watson and Brickson (2018) reported that increases in training load negatively affect sleep quality and consequently impacts athletes’ well-being. Although the authors measured sleep quality (mediator) before assessing the outcome well-being, this does not rule out that the participants may have already had a poor quality of life before any sleep alterations were assessed. In our study, only 24% of studies (*n* = 12) assessed exposure, mediator and outcome at three different time points. Vo et al. (2019) also found that less than half (47%) of studies assessed mediator and outcome at different time points in mediation analyses of randomised controlled trials. When the researchers do not have access to variables at different time points or there is the possibility of reverse causality, the causal relationship between variables depends on a theoretical plausibility that one variable precedes another and on a series of exploratory analyses to test the plausibility of the hypothesised direction of paths in the mediation model (Wiedermann & von Eye, 2015).

Interpretation and synthesis of mediation analyses depend on accurate reporting of effect size estimates (Cashin et al., 2019). A large number of included studies (52%) did not report the mediator-outcome effect (*path b* of the mediation model). Some studies did not report the direct effect (28%, *n* = 14), indirect effect (8%, *n* = 4), and the exposure-mediatior effect (*path a* of the mediation model) (12%, *n* = 6). Our findings are similar to Gelfand et al. (2009) who found that more than half of a sample of mediation studies did not report all relevant effects in the mediation model of the interest.

Five studies (10%) in our sample explicitly stated that they applied modern approaches of mediation analysis such as the counterfactual framework (Online Resource 4). Four out of five of these studies explicitly reported controlling for exposure-mediator, exposure-outcome and mediator outcome confounding and described sensitivity analysis approaches, and three out of five considered exposure-mediator interactions. A previous systematic review including studies that applied causal mediation analysis methods using the counterfactual framework described that most studies reported causal assumptions and used sensitivity analyses to address confounding (Liu et al., 2016). However, only half of those studies (6 out of 13) considered exposure-mediator interactions. From the studies that used traditional methods (*n* = 45), four studies tested the presence of exposure-mediator interaction, eight studies described the method for adjusting for confounders and two studies reported the method for sensitivity analysis. These findings suggest that the awareness of causal inference principles and modern techniques of mediation analysis may improve the quality of reporting in mediation studies.

A Guideline for Reporting Mediation Analyses (AGReMA) (Lee et al., 2021) is an evidence- and consensus-based reporting guideline recently developed using the Enhancing Quality and Transparency of Health Research (EQUATOR) methodological framework for developing reporting guidelines (Moher et al., 2010). The long-form AGReMA Statement includes 25 items across the different sections of the studies. This systematic review investigated several items from the AGReMA Statement (Online Resources 3). We identified that most studies reported terms related to “mediation analysis” in the title and abstract, described the background and theoretical rationale for investigating the mechanisms of interest in the introduction, reported the main effect of interest, included a graphic representation of the assumed causal model including the exposure, mediator and outcome but missed graphical representation of possible confounders. In addition, most studies reported how and when the variables were measured, described the statistical methods and software used to estimate the causal relationships of interest. Although studies commonly reported the point estimates for the exposure-mediator, most studies did not describe the point estimate for the mediator-outcome and the uncertainty estimates for the exposure-mediator and mediator-outcome relationships. Most studies did not report references to any protocol or study registrations specific to mediation analyses, did not report any sample size rationale, were not explicit about the assumptions of the causal model (e.g. no unmeasured confounding, temporal precedence of the variables) and did not report the presence or absence of missing data. The authors are encouraged to use AGReMA to facilitate peer review and help ensure that studies using mediation analyses are completely, accurately, and transparently reported.

### Implications for Future Work

Incomplete reporting of mediation analyses limits clinical application, reproducibility and evidence synthesis. Inadequate reporting also limits the assessment of risk of bias in studies that use mediation analyses. This limitation impedes translation of mechanistic evidence into practice and policy. We suggest that the awareness of causal inference principles and the use of a recently published guideline for reporting mediation analysis studies (AGReMA Statement) may improve the reporting of mediation analyses of observational studies in the following years.

### Limitation and Strengths

This review may not generalise to all observational studies that used mediation analysis. We selected a random sample of observational studies over the past 2 years to capture recent sample of published observational studies that used mediation analysis. We have no reason to suspect that the reporting quality of mediation studies were better or worse before the 2-year period. Although guided by previous studies, we included a modest sample of observational studies that used mediation analysis to understand mechanisms of health exposures. In addition, our interpretation may be limited by the type of study designs included in our systematic review. For example, our review did not include any case–control studies. However, we have no reason to suspect that the reporting quality of mediation studies was better or worse in case–control studies. We screened records and extracted data items in duplicate for a subset of records until satisfactory agreement was achieved. Screening and extraction were completed by a single author thereafter. We are aware that this process might cause potential mistakes, but we believe that the risk of errors did not influence our results since there was substantial agreement between reviewers (83%).

## Conclusions

Mediation analysis is a common method used to investigate the mechanisms of prevention strategies. We show that the reporting of mediation analysis in observational studies is incomplete, which can interfere with research interpretation, reproducibility, evidence synthesis and policy application. The awareness of poor reporting combined with the endorsement of a reporting guideline designed for mediation analyses may improve the standardization, transparency and completeness in the reporting of mediation studies in prevention research.

## Supplementary Information

Below is the link to the electronic supplementary material.

Supplementary file1 (DOCX 29 KB)

Supplementary file2 (DOCX 46 KB)

Supplementary file3 (DOCX 50 KB)

## Data Availability

The data set used and analysed during this study is available from the corresponding author on reasonable request.
