# Supplementary material for: A Systematic Review of the Reporting Quality of Observational Studies That Use Mediation Analyses
Source: Prev Sci. 2022 Feb 15;23(6):1041–52. doi: 10.1007/s11121-022-01349-5 (PMC9343342; doi:10.1007/s11121-022-01349-5)
Supplement: Supplementary file 1 — Supplementary file1 (DOCX 29 KB) [file 11121_2022_1349_MOESM1_ESM.docx]

# **Online Resource 2**

Search strategy: for MEDLINE (OVID) 30^th^ June 2017 to 30^th^ June 2019)

Part A: Mediation analysis terms: adapted from Lee et al. (2015).

1. Mediation analysis.mp
2. Structural equation modeling.mp
3. Structural equation modelling.mp
4. Baron and Kenny.mp
5. Product of coefficient.mp
6. Difference in coefficient.mp
7. Process of change.mp
8. Sobel$.mp
9. Causal pathway.mp
10. Indirect effect.mp
11. Process variable.mp
12. Process evaluation.mp
13. OR/1-12

# Part B: Filters

1. 13 NOT (exp animals/ not humans.sh)
2. Limit 13 to English language
3. Limit 28 to ed=20170630-20190630

(13 is all records that mention one of these terms for mediation analysis; 14 excludes animals’ experiments; 15 limits to English language; 16 limits to 30 June 2017 – 30 June 2019).

Lee, H., M. Hubscher, G. L. Moseley, S. J. Kamper, A. C. Traeger, G. Mansell & J. H. McAuley (2015) How does pain lead to disability? A systematic review and meta-analysis of mediation studies in people with back and neck pain. *Pain,* 156**,** 988-97.
