# Supplementary material for: A Systematic Review of the Reporting Quality of Observational Studies That Use Mediation Analyses
Source: Prev Sci. 2022 Feb 15;23(6):1041–52. doi: 10.1007/s11121-022-01349-5 (PMC9343342; doi:10.1007/s11121-022-01349-5)
Supplement: Supplementary file 2 — Supplementary file2 (DOCX 46 KB) [file 11121_2022_1349_MOESM2_ESM.docx]

# **Online Resource 3**

## Online Resource 3. Items included in the checklist

| **Section/ Topic** | **Reporting item** | **Options** |
| --- | --- | --- |
| **Title and abstract*** |  |  |
|  | Does the article report the mechanistic nature of the study?  We considered “yes” if authors referred in the title or abstract terms like causality, mechanisms evaluation, indirect effect and mediation analysis. | a. Title only  b. Abstract only  c. Title and Abstract  d. Not mentioned in the title and abstract  e. Unclear (we are not sure the mechanistic nature of the study in the title and abstract) |
| **Introduction** |  |  |
| Motivation | Does the article report the motivation for studying mechanisms?  (e.g. improve understanding of mechanisms, confirm or refute mechanistic theory, refine interventions or other) | Yes or no |
| Rationale* | Does the article provide a theory or evidence to support the rationale for studying the specified mechanism? | Yes or no |
| Intention | Does the article define the main intention of the study? | a. Exploratory (explore the relationship between variables)  b. Confirmatory (confirm a hypothesis for the mediation model)  c. Unclear |
| Hypothesis | Does the article include a clear hypothesis for studying mechanism? | Yes, no or unclear |
| Exposure-mediator and exposure-outcome explanation* | Does the article provide preliminary evidence or theoretical explanation for the relationship between exposure-mediator and exposure-outcome? | Yes, no or unclear |
| Mediator-outcome explanation* | Does the article provide preliminary evidence or theoretical explanation for the relationship between mediator-outcome? | Yes, no or unclear |
| **Methods** |  |  |
| Protocol or preregistration* | Does the article provide reference to a protocol or a preregistration for the mediation study? | Yes or no |
| Study design* | Does the article describe how the study design features allow for testing mechanisms? | Yes, no or unclear |
| Broad approach for mediation analysis | Does the study refer to causal mediation analysis or counterfactual framework for informing mediation analysis? | Yes or no |
| Main effect specification* | Does the article specify the main effect of interest?  (e.g. indirect effect, direct effect, controlled direct effect | Yes, no or unclear |
| Graphical representation* | Does the article include a graphical representation of the model being tested?  (e.g. directed acyclic graph, structural equation model) | Yes or no |
| Confounders (adjustment)* | Does the article consider possible confounders in the exposure-mediator, exposure-outcome, and mediator-outcome relationship? | a. Exposure-mediator and exposure outcome  b. Mediator-outcome  c. No  d. Unclear (authors say that adjusted for covariates, but they do not specify the mediation path). |
| Confounders (method for adjustment)* | Does the article describe which method was used to adjust for measured confounders? | Yes, no or unclear |
| Interaction (consideration)* | Does the article identify exposure-mediator interaction? | Yes, no or unclear |
| Interaction (modelling)* | Does the article explain *how* exposure-mediator interaction was modelled? | Yes, no or unclear |
| Nature of the variables* | When necessary, do the article specify the multilevel nature of the exposure, mediator, or outcome?  (e.g., exposure and mediator at group level, outcome at individual level) | Yes, no, unclear or not applicable |
| Assumptions* | Does the article specify the assumptions required for making causal inference? | a. no confounding of exposure-mediator effect  b. no confounding of exposure-outcome effect  c. no confounding of mediator-outcome effect  d. no exposure-dependent confounding of mediator-outcome effect  e. no interactions  f. consistency  g. positivity |
| Measurement (tool)* | Does the article state *how* the exposure, mediator and outcome were defined and measured? | Yes, no, partially or unclear |
| Measurement (time)* | Does the article state *when* the exposure, mediator and outcome were defined and measured? | Yes, no, partially or unclear |
| Sample size* | Does the article state how sample size was estimated for the mediation model | Yes, no or unclear |
| Statistical method* | Does the article specify the *statistical method* used to assess mediation?  e.g. difference-coefficient approach, product-coefficient approach, Baron and Kenny‘s framework, contrafactual-based approaches); | Yes, no or unclear |
| Statistical model* | Does the article specify the *statistical model* (functional form) used to assess mediation?  e.g. linear regression, logistic regression | Yes, no or unclear |
| Missing data (mention)* | Does the article mention missing data? | Yes or no |
| Missing data (handling)* | Does the article mention how missing data was handled? | Yes or no |
| Sensitivity analysis* | Does the article describe the approach to any sensitivity analysis used in the study? | Yes, no or unclear |
| Statistical software* | Does the article provide references to statistical software or packages used in the mediation analysis? | Yes, no or unclear |
| **Results** |  |  |
| Total effect* | Does the article provide estimates for the total effect? | a. effect  b. Precision  c. p value  d. none |
| Direct effect* | Does the article provide estimates for the direct effect? | a. effect  b. Precision  c. p value  d. none |
| Indirect effect* | Does the article provide estimates for the indirect effect? | a. effect  b. Precision  c. p value  d. none |
| Exposure-mediator effect* | Does the article provide estimates for the exposure-mediator effect? | a. effect  b. Precision  c. p value  d. none |
| Mediator-outcome effect* | Does the article provide estimates for the mediator-outcome effect? | a. effect  b. Precision  c. p value  d. none |
| Sensitivity analysis* | Does the article provide the results from sensitivity analysis?  e.g. the level of unmeasured confounding that would invalidate the effect of interest. | a. effect  b. Precision  c. p value  d. none |

Note: The items from the extraction form assessed the characteristics reported in the title, abstract, introduction, methods and results. In some items, reviewers could check “Yes” (item reported and clearly described), “No” (item not reported or not clearly described), “Unclear” (unable to discern if the item is clearly reported), “Partial” (not fully reported), or multiple choices.

* means items included in the “A Guideline for Reporting Mediation Analyses of Randomized Trials and Observational Studies” (The AGREMA Statement).
