# Supplementary material for: A Systematic Review of the Reporting Quality of Observational Studies That Use Mediation Analyses
Source: Prev Sci. 2022 Feb 15;23(6):1041–52. doi: 10.1007/s11121-022-01349-5 (PMC9343342; doi:10.1007/s11121-022-01349-5)
Supplement: Supplementary file 3 — Supplementary file3 (DOCX 50 KB) [file 11121_2022_1349_MOESM3_ESM.docx]

**Online Resource 4**

# **Online Resource 4.** Included studies

| **First author** | **Year** | **Title** | **Name of the journal** |
| --- | --- | --- | --- |
| Wang et al. | 2018 | Physical activity as a mediator of the associations between perceived environments and body mass index in Chinese adolescents | Health and Place |
| Rojo-Wissar et al., | 2019 | Sleep quality and perceived health in college undergraduates with adverse childhood experiences | Sleep Health Journal of the National Sleep Foundation |
| Haba-Rubio et al. | 2018 | Do diurnal cortisol levels mediate the association between sleep disturbances and cognitive impairment? | Neurobiology of Aging |
| Pfeffer et al. | 2018 | Behavioural automaticity moderates and mediates the relationship of trait self-control and physical activity behaviour | Psychology & Health |
| Sohn et al. | 2018 | The Relationship between Emotional Labor and Job Stress among Hospital Workers | Preventive Medicine, Occupation & Environmental Medicine |
| Luo et al. | 2018 | A Schizophrenia-Related Genetic-Brain-Cognition Pathway Revealed in a Large Chinese Population | EBioMedicine |
| Rosen et al. | 2018 | Negative voice-content as a full mediator of a relation between childhood adversity and distress ensuing from hearing voices | Schizophrenia Research |
| Garcia‑Villamisar et al. | 2019 | Internalizing Symptoms Mediate the Relation Between Acute Pain and Autism in Adults | Journal of Autism and Developmental Disorders |
| Craig et al. | 2018 | The association between self-rated health and social environments, health behaviors and health outcomes: a structural equation analysis | BMC Public Health |
| Lee et al.* | 2018 | Mechanisms of implementing public health interventions: a pooled causal mediation analysis of randomised trials | Implementation Science |
| Baoer et al. | 2018 | Depressive symptoms, post-traumatic stress symptoms and suicide risk among graduate students: The mediating influence of emotional regulatory | Psychiatry Research |
| Fuente et al. | 2018 | How Loneliness is Associated with Depressive Symptoms in Spanish College Students: Examining Specific Coping Strategies as Mediators | The Spanish Journal of Psychology |
| Gallego et al. | 2018 | Contribution of the Five Factors of Personality and Peers on Adolescent Alcohol Use: A Cross-National Study | The Spanish Journal of Psychology |
| Brinck et al. | 2018 | Inflammation functions as a key mediator in the link between ACPA and erosion development: an association study in Clinically Suspect Arthralgia | Arthritis Res Ther. |
| Kauffman et al. | 2018 | Body Mass Index and Functional Impairment: The Explanatory Role of Anxiety Sensitivity Among Treatment-seeking Smokers | Psychol Health Med |
| McCanlies et al. | 2018 | The effect of social support, gratitude, resilience and satisfaction with life on depressive symptoms among police officers following Hurricane Katrina | Int J Soc Psychiatry |
| Watson et al. | 2018 | Impaired Sleep Mediates the Negative Effects of Training Load on Subjective Well-Being in Female Youth Athletes | SPORTS HEALTH |
| Park et al. | 2018 | The relationships between timing of first childbirth, parity, and healthrelated quality of life | Quality of Life Research |
| Rusu et at. | 2017 | Economic strain and support in couple: The mediating role of positive emotions | Stress and Health |
| Mun et al. | 2018 | Adolescence effortful control as a mediator between family ecology and problematic substance use in early adulthood: A 16-year prospective study | Development and Psychopathology |
| Loenneke et al. | 2018 | Statin use may reduce lower extremity peak force via reduced engagement in muscle-strengthening activities | Clin Physiol Funct Imaging |
| Sikharulidze et al. | 2017 | Posttraumatic Stress Disorder and Somatic Complaints in a Deployed Cohort of Georgian Military Personnel: Mediating Effect of Depression and Anxiety | Journal of Traumatic Stress |
| Fareria et al. | 2017 | Altered ventral striatal-medial prefrontal cortex resting-state connectivity mediates adolescent social problems after early institutional care | Dev Psychopathol |
| Bijlsma et al.* | 2017 | Unemployment and subsequent depression: A mediation analysis using the parametric G-formula | Social Science & Medicine |
| Sancheza et al. | 2017 | Childhood Physical and Sexual Abuse Experiences Associated with Post Traumatic Stress Disorder among Pregnant Women | Ann Epidemiol |
| Shaw et al. | 2017 | Obsessive-Compulsive and Depressive Symptoms: The Role of Depressive Cognitive Styles | The Journal of Psychology |
| Short et al. | 2017 | Understanding the Relationship between Socio-Economic Status, Physical Activity and Sedentary Behaviour, and Adiposity in Young Adult South African Women Using Structural Equation Modelling | Behaviour Research and Therapy |
| Diggs et al. | 2017 | The association of harsh parenting, parent-child communication, and parental alcohol use with male alcohol use into emerging adulthood | J Adolesc Health |
| Andersen et al.* | 2017 | The indirect and direct pathways between physical fitness and academic achievement on commencement in post-compulsory education in a historical cohort of Danish school youth | BMC Public Health |
| Liu et al. | 2017 | Blood monocyte transcriptome and epigenome analyses reveal loci associated with human atherosclerosis | nature communications |
| Short et al. | 2017 | Sleep disturbance as a predictor of affective functioning and symptom severity among individuals with PTSD: An ecological momentary assessment study | Behaviour Research and Therapy |
| Tsur et al. | 2017 | Torturing personification of chronic pain among torture survivors | Journal of Psychosomatic Research |
| Balogun et al | 2017 | Maternal education and child immunization: the mediating roles of maternal literacy and socioeconomic status | Pan African Medical Journal |
| Smedema SM | 2017 | Evaluation of a Concentric Biopsychosocial Model of Well-Being in Persons With Spinal Cord Injuries | Rehabilitation Psychology |
| Carcelen et al. | 2017 | Behavioral Problems and Socioemotional Competence at 18 to 22 Months of Extremely Premature Children | PEDIATRICS |
| Bal et al.* | 2017 | Does Psychosocial Stress Explain Socioeconomic Inequities in 9-Year Weight Gain Among Young Women? | Obesity |
| Kelly et al. | 2017 | Substituting activities mediates the effect of cognitive flexibility on physical activity: a daily diary study | J Behav Med |
| Comulada et al. | 2017 | Factors Related to Client Satisfaction with Methadone Maintenance Treatment in China | J Subst Abuse Treat |
| Ding et al. | 2017 | Multidimensional predictors of physical frailty in older people: identifying how and for whom they exert their effects | Biogerontology |
| Fujishiro et al.* | 2017 | ‘Doing what I do best’: The association between skill utilization and employee health with healthy behavior as a mediator | Social Science & Medicine |
| Chew et al. | 2017 | Individual and family factors associated with self-esteemin young people with epilepsy: A multiple mediation analysis | Epilepsy & Behavior |
| Wang et al. | 2017 | Teachers’ goal orientations: Effects on classroom goal structures and emotions | British Journal of Educational Psychology |
| Burholt et al. | 2017 | A Social Model of Loneliness: The Roles of Disability, Social Resources, and Cognitive Impairment | The Gerontologist |
| Choi et al. | 2017 | Mental health and health-related quality of life of Chinese college students who were the victims of dating violence | Qual Life Res |
| Panuwatwanich et al. | 2017 | Influence of safety motivation and climate on safety behaviour and outcomes: evidence from the Saudi Arabian construction industry | International Journal of Occupational Safety and Ergonomics |
| DeCou et al. | 2017 | Assault-Related Shame Mediates the Association Between Negative Social Reactions to Disclosure of Sexual Assault and Psychological Distress | Psychological Trauma: Theory, Research, Practice, and Policy |
| Hou et al. | 2017 | Parents’ Perceived Discrimination and Adolescent Adjustment in Chinese American Families: Mediating Family Processes | Child Development |
| Hermetet-Lindsay et al. | 2017 | Contributions of Disease Severity, Psychosocial Factors, and Cognition to Behavioral Functioning in US Youth Perinatally Exposed to HIV | AIDS Behav. |
| Chin et al. | 2017 | Cognition and Health Literacy in Older Adults’ Recall of Self-Care Information | The Gerontologist |
| Su et al. | 2018 | Influence of Parental Alcohol Dependence Symptoms and Parenting on Adolescent Risky Drinking and Conduct Problems: A Family Systems Perspective | Alcohol Clin Exp Res |

* Studies that used the counterfactual approach for mediation analysis.
